# One-Year Morbidity Following Videoscopic Inguinal Lymphadenectomy for Stage III Melanoma

**DOI:** 10.3390/cancers13061450

**Published:** 2021-03-22

**Authors:** Marnix R. Jansen, Otis M. Vrielink, Marloes Faut, Eric A. Deckers, Lukas B. Been, Barbara L. van Leeuwen

**Affiliations:** Department of Surgical Oncology, University Medical Center Groningen, University of Groningen, Hanzeplein 1, P.O. Box 30.001, 9700RB Groningen, The Netherlands; m.r.jansen@umcg.nl (M.R.J.); o.m.vrielink@umcg.nl (O.M.V.); m.faut@umcg.nl (M.F.); e.a.deckers@umcg.nl (E.A.D.); l.b.been@umcg.nl (L.B.B.)

**Keywords:** melanoma, inguinal metastases, videoscopic inguinal lymphadenectomy, morbidity, lymphedema, quality of life

## Abstract

**Simple Summary:**

Inguinal lymphadenectomy (the removal of lymph nodes in the groin) is currently part of the treatment options for stage III melanoma patients. Surgery can be performed using one large inguinal incision (open approach) or a few smaller incisions (videoscopic approach). Previous research has already shown less severe complications and comparable oncologic outcomes after the videoscopic approach. Postoperative lymphedema following inguinal lymphadenectomy is a well-known problem which can potentially decrease quality of life. With the arrival of adjuvant systemic treatment options, less invalidating surgery is highly desirable. However, lymphedema and quality of life have only been investigated after the open approach. Therefore, we evaluated lymphedema and quality of life following videoscopic inguinal lymphadenectomy for stage III melanoma. The videoscopic inguinal lymphadenectomy is a feasible approach due to the comparable lymphedema incidence and normalization of quality of life during follow-up.

**Abstract:**

Purpose: We aimed to elucidate morbidity following videoscopic inguinal lymphadenectomy for stage III melanoma. Methods: Melanoma patients who underwent a videoscopic inguinal lymphadenectomy between November 2015 and May 2019 were included. The measured outcomes were lymphedema and quality of life. Patients were reviewed one day prior to surgery and postoperatively every 3 months for one year. Results: A total number of 34 patients were included for participation; 19 (55.9%) patients underwent a concomitant iliac lymphadenectomy. Lymphedema incidence was 40% at 3 months and 50% at 12 months after surgery. Mean interlimb volume difference increased steadily from 1.8% at baseline to 6.9% at 12 months (*p* = 0.041). Median Lymph-ICF-LL total score increased from 0.0 at baseline to 12.0 at 3 months, and declined to 8.5 at 12 months (*p* = 0.007). Twelve months after surgery, Lymph-ICF-LL scores were higher for females (*p* = 0.021) and patients that received adjuvant radiotherapy (*p* = 0.013). The Median Distress Thermometer and EORTC QLQ-C30 summary score recovered to baseline at 12 months postoperatively (*p* = 0.747 and *p* = 0.203, respectively). Conclusions: The onset of lymphedema is rapid and continues to increase up to one year after videoscopic inguinal lymphadenectomy. Quality of life recovers to the baseline value.

## 1. Introduction

Cutaneous melanoma is a potentially life-threatening disease, and the incidence is increasing rapidly across the globe [1]. Melanoma of the lower extremity and lower trunk can spread to draining lymph nodes in the groin. As with other malignancies, lymph node status is a powerful predictor of recurrence and survival [2]. Inguinal Lymphadenectomy (IL) consists of the en bloc resection of fibrofatty tissue within the femoral triangle and has long been the standard of care for regionally metastatic melanoma. However, treatment for inguinal metastatic melanoma is currently changing. Routine completion IL does not improve overall or melanoma-specific survival and is, therefore, no longer warranted. In contrast, a therapeutic IL remains part of the treatment options for clinically detected nodal metastases [3,4].

Open inguinal lymphadenectomy (OIL) is accompanied by a high incidence of wound-related postoperative complications, occurring in up to 70% of patients [5,6,7,8,9,10,11,12,13]. The large inguinal incision potentially causes complications such as infection, dehiscence and necrosis. Videoscopic inguinal lymphadenectomy (VIL) is a minimally invasive technique designed to diminish these complications. Since VIL is associated with comparable but less extensive postoperative complications and acceptable oncologic outcome, it is considered a feasible alternative to the traditional approach [14,15,16,17,18,19,20,21,22]. In addition to wound-related complications, postoperative lymphedema is also a cause for concern for both patients and clinicians. Lymphedema is reflected in enlarged extremities, mobility problems and regional symptoms. Up to 72% of patients experience lymphedema following OIL [5,7,11,12,23,24,25]. Lymphedema could reduce quality of life (QoL) due to lymphedema-related complaints, restrictions in daily activities and decreased cosmetic satisfaction. However, data regarding morbidity in terms of lymphedema and QoL after VIL for metastatic melanoma are scarce.

The purpose of this study is to provide new insights into the morbidity after VIL for stage III melanoma during a one-year follow-up.

## 2. Materials and Methods

A prospective cohort study was performed at the University Medical Center Groningen (UMCG), a tertiary referral center for melanoma in the north of the Netherlands. The patient eligibility included histopathologically confirmed inguinal metastases from melanoma (micrometastases)—by sentinel lymph node biopsy (SLNB) or fine-needle aspiration—or clinically palpable lymph nodes (macrometastases). The surgical procedure consisted of VIL, in some cases followed by open iliac lymphadenectomy via separate skin incision. All consecutive melanoma patients requiring VIL between November 2015 and May 2019 were included. Patients with a history of surgery to the groin (except SLNB) were excluded.

### 2.1. Outcomes and Measurements

Baseline characteristics, i.e., the patient’s age, gender, Body Mass Index (BMI), medical history and histopathological, surgical and postoperative characteristics, were obtained from electronic medical files. The outcomes consisted of the incidence and course of morbidity in terms of lymphedema (volumetry measurements and Lymph-ICF-LL questionnaire) and QoL (Distress Thermometer and Problem List, EORTC QLQ-C30 questionnaire and Body Image Scale). Patients were reviewed one day prior to surgery and postoperatively every 3 months for one year. At each visit, limb volume was measured, and patients filled out the four questionnaires mentioned above.

Concerning the lymphedema-related disability, volumetry measurements—determined by a water displacement technique [26]—and the Lymph-ICF-LL questionnaire [27] were conducted. Limb length up to the groin was measured for each patient individually. Patients positioned their legs in a water-filled cylinder up to the measured limb length. The displaced water volume was measured separately for both legs. The interlimb volume difference was computed by subtracting the volume of the contralateral leg from the volume of the operated leg. The interlimb volume difference was expressed as a percentage, calculated by dividing the interlimb volume difference by the volume of the contralateral leg. A cut-off value of 6.5% was used to determine lymphedema incidence [7]. The Lymph-ICF-LL questionnaire is frequently used to evaluate patients with lower limb lymphedema. It comprises 28 questions on function decline, activity limitations and participation restrictions. The total Lymph-ICF-LL score ranges from 0 to 100, and higher scores indicate more disability. Lymphedema is classified as a Lymph-ICF-LL score > 5 [27]. To quantify the systemic response to the videoscopic approach, plasma levels of C-reactive protein (CRP) and lactate dehydrogenase (LDH) were determined at baseline and two days postoperatively. Differences in CRP and LDH were calculated for each patient.

The Distress Thermometer (DT) and Problem List (PL) [28] are Dutch tools commonly used to evaluate distress in oncological patients. The DT was used to monitor overall distress during the week prior to measurement. The DT score ranges from 0 to 10, no to extreme distress. The PL consists of 47 questions, further demonstrating distress on practical, psychosocial or physical grounds. The total PL score ranges from 0 to 47, and higher scores signify increased distress. The EORTC QLQ-C30 [29] is among the most widely used quality of life questionnaires in cancer research. The EORTC QLQ-C30 summary score, calculated from 13 of the 15 EORTC QLQ-C30 scales, and the EORTC QLQ-C30 QoL score were used. Both measures range from a score of 0 to 100. A higher EORTC QLQ-C30 summary score denotes a better level of overall functioning; an elevated EORTC QLQ-C30 QoL score indicates a greater quality of life. The Body Image Scale (BIS) [30,31] is used to assess body image and any changes in body image after cancer diagnosis or treatment. The total BIS score ranges from 0 to 30, higher scores implying inferior body image.

### 2.2. Surgical Procedure

The surgical procedure has already been described thoroughly in the previous literature [18,32]. Surgery was performed as reported in preceding research [17] and is briefly summarized below. Patients were positioned on a split-leg table. The operative limb was externally rotated and placed in abduction. The surgeon was positioned between the patient’s legs, whilst the assistant stood laterally to the operative limb. The boundaries of the femoral triangle were designed, and incision sites were marked. The femoral triangle was insufflated up to 25 mmHg under visualization with a thirty degree scope. A triangulated 3-trocar technique was used to dissect the specimen. Dissection was performed within the femoral triangle superficial to Scarpa’s fascia. The lymph node packet was placed in a removal bag and extracted through the apical incision site. A drain was left behind in the medial incision site. In the event of a concomitant open iliac lymphadenectomy, entrance to the deep pelvis was gained via a separate skin incision. The dissection was executed along the external iliac artery. Postoperatively, drains were removed if production fell below 50 mL/day. Patients could mobilize immediately and after 24 h in case of a concomitant iliac lymphadenectomy.

### 2.3. Statistical Analysis

Baseline characteristics and complications were described as the count with percentage (%) for categorical variables and as the median with range for continuous variables. The interlimb volume difference and total scores from previously mentioned questionnaires were presented as the mean with standard deviation (SD) when normally distributed and as the median with interquartile range (IQR) when skewedly distributed. To compare the baseline with 12 months of follow-up measures, the dependent samples *t*-test and Wilcoxon signed-rank test were performed. The Mann–Whitney U test was used to compare Lymph-ICF-LL, DT and PL scores for patients with or without objectively measured lymphedema at 12 months after surgery. The association between independent variables and lymphedema was assessed by univariable linear regression. The following variables were analyzed for their potential association with lymphedema based on the literature [23,33]: gender, BMI (<25 or ≥25 kg/m^2^), surgical procedure (with or without iliac lymphadenectomy) and adjuvant radiotherapy. Mean differences of CRP and LDH for patients with and without lymphedema at 12 months postoperatively were compared using an unpaired *t*-test. SPSS Statistics, version 23 (IBM Corp. Released 2013. IBM SPSS Statistics for Windows, Version 23.0. Armonk, NY, USA: IBM Corp.), was used for statistical analysis, and *p* < 0.05 was considered as statistically significant.

### 2.4. Ethical Approval

Data collection was conducted in accordance with the declaration of Helsinki [34] and its later amendments. The Medical Ethical Committee granted dispensation for the Dutch law regarding patient-based medical research (WMO) obligation (METc registration no 2015279).

## 3. Results

Baseline characteristics are presented in Table 1. Between November 2015 and May 2019, a total number of 34 patients were included for participation. The median patient age was 56 years (range: 29–78); 24 (70.6%) were females. Superficial spreading melanoma (55.9%) was the most frequently reported histological subtype, followed by nodular melanoma (20.6%). The median Breslow thickness was 2.5 mm (range: 0.3–10.0), and 10 (29.4%) melanomas showed ulceration. The median BMI was 26.5 kg/m^2^ (range: 18.0–43.1). Two (5.9%) patients suffered from diabetes mellitus, and six (17.6%) patients had a history of smoking. Thirteen (38.2%) patients underwent surgery for micrometastases, and 19 (61.8%) patients for macrometastases. The median nodal yield following VIL was 8.5 (range: 1–19). A concomitant iliac lymphadenectomy was performed in 19 (55.9%) patients. One procedure was converted because of fibrosis and seroma after prior diagnostic lymph node excision. This patient was included based on the intention-to-treat principle. Postoperatively, 10 (29.4%) patients underwent radiotherapy. Wound complications occurred in 24 patients (70.6%). Seroma was reported most frequently (50%), followed by wound infection (29.4%). All complications were treated conservatively, including seroma aspiration and oral antibiotics.

### 3.1. Lymphedema

**Figure 1:**
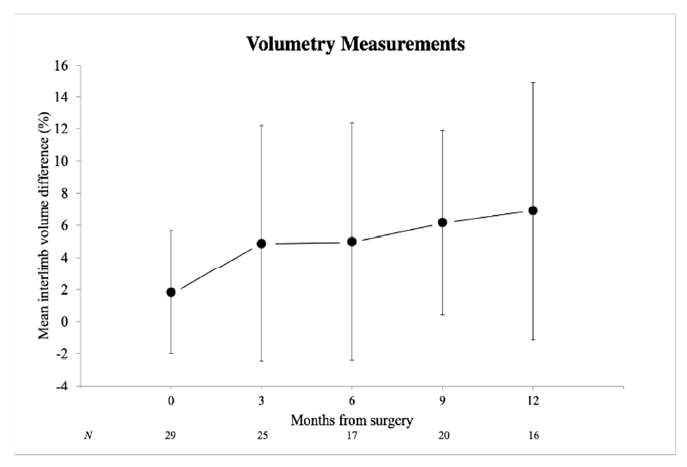
Volumetry measurements in mean interlimb volume difference (%) at individual time points. Error bars represent standard deviation of the mean.

Lymphedema-related morbidity was measured both objectively and subjectively for all 34 patients. Objectively measured lymphedema incidence was 10.3% at baseline, 40.0% at 3 months, 35.3% at 6 months, 40.0% at 9 months and 50.0% at 12 months postoperatively. Only slight (an interlimb volume difference of 6.5–20.0%) and moderate (an interlimb volume difference of 20.1–40.0%) edema was reported. The mean interlimb volume difference was 1.8% at baseline. The mean interlimb volume difference increased after surgery to 4.9%, 5.0%, 6.2% and 6.9% at 3, 6, 9 and 12 months, respectively (Figure 1). At 12 months after surgery, the mean interlimb volume difference was evidently higher compared to the baseline value (*p* = 0.041) (Table 2). Patients with objectively measured lymphedema (an interlimb volume difference >6.5%) at 12 months following surgery did not have significantly higher Lymph-ICF-LL (*p* = 0.916), DT (*p* = 0.844) or PL (*p* = 0.512) scores. Univariable linear regression analysis did not show an association between interlimb volume difference at 12 months after surgery and gender (*p* = 0.230), BMI (*p* = 0.619), surgical procedure (*p* = 0.475) or adjuvant radiotherapy (*p* = 0.991) (Table 3). The mean difference (SD) CRP values were 32.5 (46.5) mg/L for patients with lymphedema and 39.1 (40.5) mg/L for patients without lymphedema (*p* = 0.831). The mean difference (SD) LDH values were −47.7 (40.9) U/L for patients with lymphedema and −41.0 (32.6) U/L for patients without lymphedema (*p* = 0.761). Thus, postoperative systemic response is not associated with lymphedema formation.

**Figure 2:**
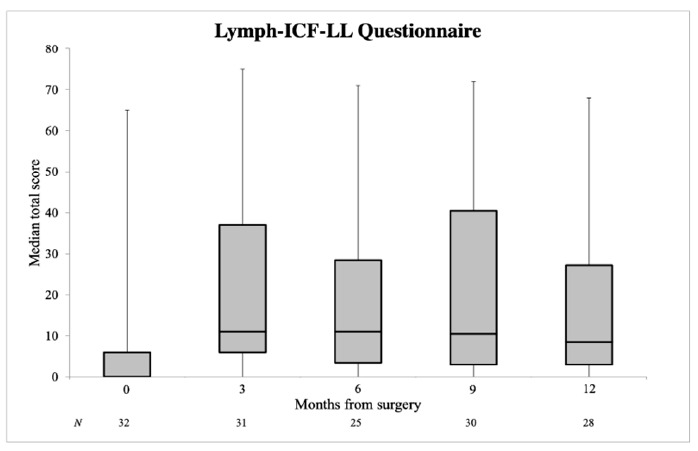
Lymph-ICF-LL questionnaire in median total score with interquartile range (IQR) at individual time points. Error bars represent minimum and maximum values. A higher Lymph-ICF-LL total score indicates more lymphedema-related disability.

Subjectively measured lymphedema incidence was 31.2% at baseline, 80.6% at 3 months, 72.0% at 6 months, 66.7% at 9 months and 71.4% at 12 months after surgery. A small lymphedema problem (a Lymph-ICF-LL total score of 5–24) occurred most frequently. The median Lymph-ICF-LL total score showed an increase at 3 months follow-up, from 0.0 preoperatively to 12.0 at 3 months. Subsequent months revealed a gradual decline in the median Lymph-ICF-LL total score: 11.0 at 6 months, 10.5 at 9 months and 8.5 at 12 months (Figure 2). The median Lymph-ICF-LL total score was significantly higher at 12 months after surgery compared to the baseline value (*p* = 0.007) (Table 2). Interestingly, at 12 months after surgery, 8 out of 16 (50.0%) patients did not have objectively measured lymphedema (an interlimb volume difference > 6.5%), whilst 6 of these 8 patients (75%) reported lymphedema subjectively (a Lymph-ICF-LL total score > 5). Univariable linear regression analysis showed higher Lymph-ICF-LL scores for females (*p* = 0.021) and patients that received adjuvant radiotherapy (*p* = 0.013). There was no association with BMI (*p* = 0.931) or surgical procedure (*p* = 0.198) (Table 4).

### 3.2. Quality of Life

**Figure 3:**
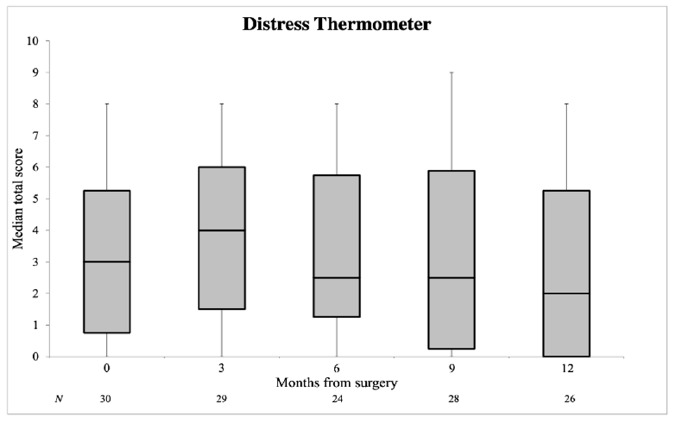
Distress Thermometer in median total score with IQR at individual time points. Error bars represent minimum and maximum values. A higher Distress Thermometer (DT) score signifies increased distress.

**Figure 4:**
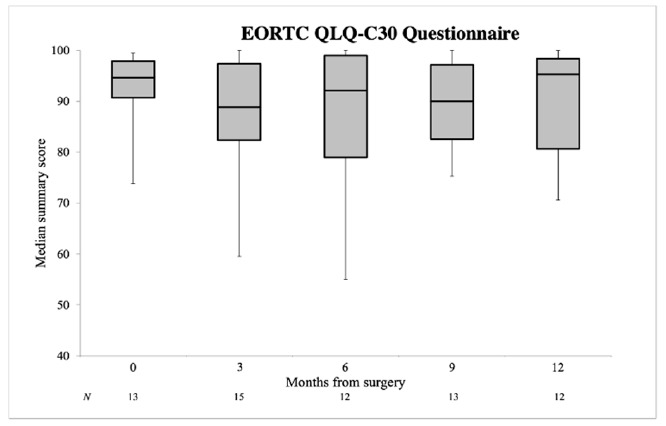
EORTC QLQ-C30 questionnaire in median summary score with IQR at individual time points. Error bars represent minimum and maximum values.

The DT and PL were obtained in 34 patients. The median DT score increased from 3.0 before surgery to 4.0 at 3 months after surgery and eventually decreased below the baseline value, namely, 2.5 at both 6 and 9 months and 2.0 at 12 months postoperatively (Figure 3). The median PL score was 4.0 at baseline, 3.0 at 3 months, 2.0 at 6 months, 4.0 at 9 months and 3.5 at 12 months. Both the median DT and PL scores were not significantly higher at 12 months after surgery compared to the baseline value (*p* = 0.747, *p* = 0.362, respectively) (Table 2). Sixteen patients completed the EORTC QLQ-C30 questionnaire. The median summary score was 94.7 at baseline and decreased to 88.8 at 3 months. In the subsequent months, the median summary score recovered to the baseline value: 95.3 at 12 months (Figure 4). The median QoL score showed a decline from 91.6 at baseline to 83.3 at 3 months and remained stable for the remainder of the study. At 12 months after surgery, both the median EORTC summary score and QoL score were not significantly lower compared to the baseline value (*p* = 0.203, *p* = 0.206, respectively) (Table 2).

The BIS was assessed in 16 patients. The median BIS was 0.0 at baseline, 2.0 at 3 months, 0.0 at 6 months, 1.0 at 9 months and 0.0 at 12 months. The BIS score thereby remained approximately equivalent to the baseline score, fluctuating between 0.0 and 2.0. The median BIS was not significantly higher at 12 months after surgery compared to the baseline value (*p* = 0.400) (Table 2).

## 4. Discussion

This prospective cohort study aimed to evaluate morbidity after VIL for patients with inguinal metastatic melanoma. Postoperative lymphedema incidence remained substantial as limb volume increased rapidly in the immediate postoperative phase and continued to increase up to 12 months after VIL. In contrast, QoL improved to baseline values during follow-up. Women and patients that underwent adjuvant radiotherapy experienced more lymphedema-related complaints at 12 months after VIL.

### 4.1. Lymphedema

In the literature, objectively measured lymphedema incidence is reported in 25%–72% of patients following OIL, depending on the definition for lymphedema used [5,7,11,12,23,24,25]. We reported a lymphedema incidence of 40% at 3 months and 50% at 12 months following VIL. Studies investigating lymphedema following OIL used measurements at different time points with a wide variety of assessment methods. In our study, the interlimb volume difference was determined using a water displacement technique. Three months after OIL, interlimb volume differences of approximately 7% to 8% were described [23,33]. We demonstrated an interlimb volume difference of 5% at 3 months following VIL. Preservation of the dermal lymphatic system and minor wound infections could explain this lower interlimb volume difference with a videoscopic approach. The interlimb volume difference increased to approximately 7% at 12 months after VIL, a phenomenon also noted in previous research regarding OIL [23]. Elevated BMI (≥25 kg/m^2^) and adjuvant radiotherapy were associated with both increased and prolonged lower limb swelling after OIL [23,33]; however, this relation was not established in the current study. Moreover, this study has shown that a concomitant iliac lymphadenectomy did not contribute to lymphedema formation. Objectively measured lymphedema at 12 months following VIL was not associated with lymphedema-related complaints (Lymph-ICF-LL questionnaire) or QoL (Distress Thermometer and Problem List). Intriguingly, the majority of patients without objectively measured lymphedema reported lymphedema subjectively at 12 months follow-up. This indicates an incongruity for the lymphedema incidence cut-off values of both assessments [7,27]. In summary, lymphedema establishes rapidly and increases further in the subsequent months after VIL. Although the interlimb volume difference seemed lower, lymphedema incidence appeared within the range described following the open approach. This is not surprising, since nodal yield is similar for both techniques [14,16,17,18,19,20,22].

### 4.2. Quality of Life

Our initial results showed a slight decrease in QoL (determined by DT, PL and EORTC QLQ-C30 scores) at 3 months postoperatively. Earlier bladder catheter removal, less pain, rapid mobilization, reduced hospitalization and earlier resumption of daily activities have been reported previously in favor of the videoscopic technique [15]. During follow-up, most QoL measures eventually recovered to baseline values. Following OIL, elevated BMI (≥25 kg/m^2^) has been associated with worse QoL and prolonged recovery [23]. The gradual restoration of QoL in the first 12 months after VIL [14] and OIL [23] has been described earlier, probably due to an acceptance of limitations in daily activities after confrontation with a life-threatening illness [6,23,35].

### 4.3. Implications

Although both the DeCOG-SLT study [3] and the MSLT-II study [4] did not prove any significant survival advancements when comparing routine completion IL to nodal observation alone, a therapeutic IL is currently considered the standard of care for clinically detectable metastases to the inguinal lymph node basin. The appropriate treatment for inguinal metastatic melanoma has gained increasing interest in recent years. Contemporary research with the use of multispectral optoacoustic tomographic imaging may allow the nonradioactive detection of sentinel lymph nodes and thereby eventually replace the SLNB [36]. Other recent developments in the treatment of inguinal metastatic melanoma concern adjuvant immunotherapy and molecular targeted therapy [37,38,39,40]. Adjuvant therapies potentially increase life expectancy for stage III melanoma patients. Minimizing morbidity following IL is extremely important in order to be eligible for adjuvant treatment. Previous research has already shown less severe complications and earlier postoperative recovery after VIL [14,15,16,17,18,19,20,21,22]. Prior to this study, evidence regarding morbidity after VIL was limited. The current study unveiled acceptable morbidity during one-year follow-up. Especially in the developing era of new adjuvant treatment options, the VIL seems an attractive alternative to the OIL for stage III melanoma.

## 5. Conclusions

The onset of lymphedema is rapid and continues to increase up to one year after VIL. During follow-up, QoL recovers to the baseline value. Although postoperative lymphedema remains a problem, with less severe complications and adequate oncologic outcomes from previous research, the VIL for stage III melanoma is a feasible alternative to the open approach.

## Figures and Tables

**Table 1:** Baseline characteristics (*n* = 34).

Patient and Melanoma Characteristics	
Gender	
Male	10 (29.4%)
Female	24 (70.6%)
Age, years ^a^	56 (29–78)
BMI, kg/m^2^	26.5 (18.0–43.1)
Diabetes Mellitus	
Yes	2 (5.9%)
No	32 (94.1%)
Smoking	
Yes	6 (17.6%)
No	28 (82.4%)
Histologic typing	
Superficial spreading	19 (55.9%)
Nodular	7 (20.6%)
Other	3 (8.8%)
Unknown primary	3 (8.8%)
Unknown	2 (5.9%)
Breslow thickness, mm	2.5 (0.3–10.0)
T stage	
Tis	2 (5.9%)
T1 (≤1.00 mm)	3 (8.8%)
T2 (1.01–2.00 mm)	9 (26.5%)
T3 (2.01–4.00 mm)	10 (29.4%)
T4 (>4.00 mm)	7 (20.6%)
Unknown primary	3 (8.8%)
Ulceration	
Yes	10 (29.4%)
No	19 (55.9%)
Unknown primary	3 (8.8%)
Unknown	2 (5.9%)
**Surgical and Postoperative Characteristics**	
Operation	
Videoscopic inguinal lymphadenectomy	15 (44.1%)
Additional open lymphadenectomy	19 (55.9%)
Indication	
Micrometastases	13 (38.2%)
Macrometastases	21 (61.8%)
Wound complication	
Yes	24 (70.6%)
No	10 (29.4%)
Adjuvant radiotherapy ^b^	
Yes	10 (29.4%)
No	24 (70.6%)

Data are presented as n (%) or median (range). Abbreviations: BMI, Body Mass Index. ^a^ Age at lymph node dissection. ^b^ Within 30 days after surgery.

**Table 2:** Lymphedema and quality of life measurements at baseline and 12 months after surgery.

Variable	Baseline	12 months	*p*-Value
Interlimb volume difference (%)	1.8 (3.8)	6.9 (8.1)	0.041
Lymph-ICF-LL	0.0 (0.0–6.0)	8.5 (3.0–27.3)	0.007
Distress Thermometer	3.0 (0.8–5.3)	2.0 (0.0–5.3)	0.747
Problem List	4.0 (1.0–6.0)	3.5 (0.0–10.5)	0.362
EORTC summary score	94.7 (90.7–97.9)	95.3 (80.6–98.3)	0.203
EORTC quality of life score	91.6 (83.3–100.0)	83.3 (75.0–100.0)	0.206
Body Image Scale	0.0 (0.0–2.0)	0.0 (0.0–5.75)	0.400

Data are presented as mean (SD) or median (IQR). Baseline scores and 12 months follow-up measures were compared with paired samples t-test when normally distributed and with Wilcoxon signed-rank test when skewedly distributed.

**Table 3:** Univariable linear regression analysis for interlimb volume difference at 12 months after surgery.

Variable	Mean Interlimb Volume Difference in %	B (95% CI)	*p*-Value
Gender			
Men	10.6 (12.2)	5.4 (−3.8–14.5)	0.230
Women	5.2 (5.3)	Reference	
BMI			
<25 kg/m^2^	7.8 (10.3)	Reference	
≥25 kg/m^2^	5.7 (4.1)	−2.1 (−11.1–6.8)	0.619
Surgical procedure			
Inguinal	8.4 (9.9)	Reference	
Iliac and inguinal	5.4 (6.0)	−3.0 (−11.8–5.8)	0.475
Adjuvant radiotherapy			
Yes	7.0 (3.6)	0.1 (−11.4–11.5)	0.991
No	6.9 (8.9)	Reference	

**Table 4:** Univariable linear regression analysis for Lymph-ICF-LL total score at 12 months after surgery.

Variable	Median Lymph-ICF-LL Total Score (IQR)	B (95% CI)	*p*-Value
Gender			
Men	4.0 (0.75–6.75)	−20.0 (−36.7–−3.2)	0.021
Women	18.00 (6.0–50.25)	Reference	
BMI			
<25 kg/m^2^	14.0 (2.0–28.0)	Reference	
≥25 kg/m^2^	7.0 (3.0–35.0)	0.7 (−16.1–17.6)	0.931
Surgical procedure			
Inguinal	6.0 (2.5–23.0)	Reference	
Iliac and inguinal	14.0 (6.0–52.0)	10.5 (−5.8–26.8)	0.198
Adjuvant radiotherapy			
Yes	45.0 (7.0–57.0)	22.3 (5.1–39.5)	0.013
No	6.0 (2.5–18.0)	Reference	

## Data Availability

The data presented in this study are available on request from the corresponding author.
